# PET microplastics affect human gut microbiota communities during simulated gastrointestinal digestion, first evidence of plausible polymer biodegradation during human digestion

**DOI:** 10.1038/s41598-021-04489-w

**Published:** 2022-01-11

**Authors:** Alba Tamargo, Natalia Molinero, Julián J. Reinosa, Victor Alcolea-Rodriguez, Raquel Portela, Miguel A. Bañares, Jose F. Fernández, M. Victoria Moreno-Arribas

**Affiliations:** 1 Institute of Food Science Research, CIAL, CSIC-UAM, c/Nicolás Cabrera, 9, 28049 Madrid, Spain; 2 Instituto de Cerámica y Vidrio, CSIC, c/Kelsen, 5, 28049 Madrid, Spain; 3 Encapsulae S.L, c/Lituania 10, 12006 Castellón de la Plana, Spain; 4 Institute of Catalysis and Petrochemistry, CSIC, C/Marie Curie, 2, 28049 Madrid, Spain

**Keywords:** Microbiology, Risk factors

## Abstract

Microplastics (MPs) are a widely recognized global problem due to their prevalence in natural environments and the food chain. However, the impact of microplastics on human microbiota and their possible biotransformation in the gastrointestinal tract have not been well reported. To evaluate the potential risks of microplastics at the digestive level, completely passing a single dose of polyethylene terephthalate (PET) through the gastrointestinal tract was simulated by combining a harmonized static model and the dynamic gastrointestinal simgi model, which recreates the different regions of the digestive tract in physiological conditions. PET MPs started several biotransformations in the gastrointestinal tract and, at the colon, appeared to be structurally different from the original particles. We report that the feeding with microplastics alters human microbial colonic community composition and hypothesize that some members of the colonic microbiota could adhere to MPs surface promoting the formation of biofilms. The work presented here indicates that microplastics are indeed capable of digestive-level health effects. Considering this evidence and the increasing exposure to microplastics in consumer foods and beverages, the impact of plastics on the functionality of the gut microbiome and their potential biodegradation through digestion and intestinal bacteria merits critical investigation.

## Introduction

The prevalence of plastic particles in the food chain^1–4^ has raised concern about the health effects of microplastics ingestion. However, the risk assessment of microplastics’ intake is still a global challenge for the scientific community^2,5–7^. Microplastics (MPs) are plastic particles with micrometer size that can be intentionally manufactured (primary MPs) or released to the environment from larger plastics (secondary MPs). The latter are able to act as vectors for pathogens^8^ and are referred to as a “cocktail of contaminants” due to their association with heavy metals, plastic additives or other persistent organic pollutants^3^. The most common polymers in Europe are, in decreasing order of production, polyethylene (PE), polypropylene (PP), polyvinyl chloride (PVC), polyurethane (PUR), and polyethylene terephthalate (PET)^9^. Among them, PET is one of the main polymers used for plastic bottles and caps, and has been detected in beverages like soft drinks^10^ (probably due to bottle degradation, although MP particles have also been detected in beer^10^ and mineral waters from glass bottles^7^).

PET is made from petroleum-derived terephthalic acid and ethylene glycol produced by melt-phase condensation and further solid-state polymerization. The presence of PET particles in human feces observed in a prospective pilot study suggests their active interaction with the human digestive system^11^. However, their changes during gastrointestinal digestion or colonic fermentation are scarcely explored. To date, only Stock et al. have simulated in vitro human digestion of several MP particles along the gastrointestinal tract^12^. They did not observe pronounced structural changes due to digestion fluids except for the formation of a surface organic corona around the MP particles. However, this recent study did not explore the colonic stage or MP interactions with colonic microbiota^12^. The primary health effects of food-ingested plastic particles are triggered from the digestive system, causing direct damage not only at local level, as irritation or intestinal dysbiosis, but also at systemic level^4^. In this regard, Fackelmann and Sommer reviewed the link between MP-induced gut dysbiosis and host health and suggested that MPs could impact gut microbiome and that chronic exposition could lead to gut dysbiosis in several species^13^. Still, the effect of microplastics on the human gut microbiome is uncertain. Few studies have investigated this issue, just in animal models, and with much higher MPs doses than those detected in edible foods and beverages^14–16^. Their most common observation has been that exposure to MPs altered gut bacterial diversity and caused other health negative effects, including changes in gut metabolic profiles and inflammation.

The human digestion of MPs and their effects on colonic microbiota can be studied with different approaches, including in vitro and in vivo approaches. Due to their physiological relevance, investigations with animals and human trials generally offer the most precise results and are still considered the ‘gold standard’ for certain diet-related questions. However, due to ethical restrictions, high cost, and the complexity of the multistage processes of human digestion, there is a real need for in vitro models that mimic the physiological conditions of human digestion. Static models can be used to determine endpoints of digestion or kinetics of very specific steps of digestion, such as stomach and small intestinal biotransformations. In contrast, dynamic models are more complex but closer to the physiological reality of the gastrointestinal tract^17,18^. One of these dynamic simulators is the simgi® system, a computer-controlled gastrointestinal in vitro model designed to reproduce the colonic microbiota responsible for metabolic bioconversions in the large intestine that has been employed to study the effect of different foods^19–25^. In addition, dynamic simulators like simgi® have been shown to be also useful to evaluate the effects on intestinal microbiota of Active Pharmaceutical Ingredients (API)^26^, heavy metals^27,28^ and food-use nanomaterials^29,30^.

In this work, PET MPs in amounts that were in the range of realistic human exposure levels of ingestion were subjected to digestion simulation in a standardized in vitro static model and to gut-microbial dynamic fermentation using the simgi® model, with the aim of providing scientific evidence of modifications and potential effects of food MPs during their passage through the digestive tract. Furthermore, changes in microbiota composition and in PET morphology were evaluated after upper static digestion and after colonic fermentation in the different simgi® colonic compartments (ascending, transverse and descending colon).

## Results

### Effect of gastrointestinal digestion and colonic fermentation in microplastics morphology

**Figure 1:**
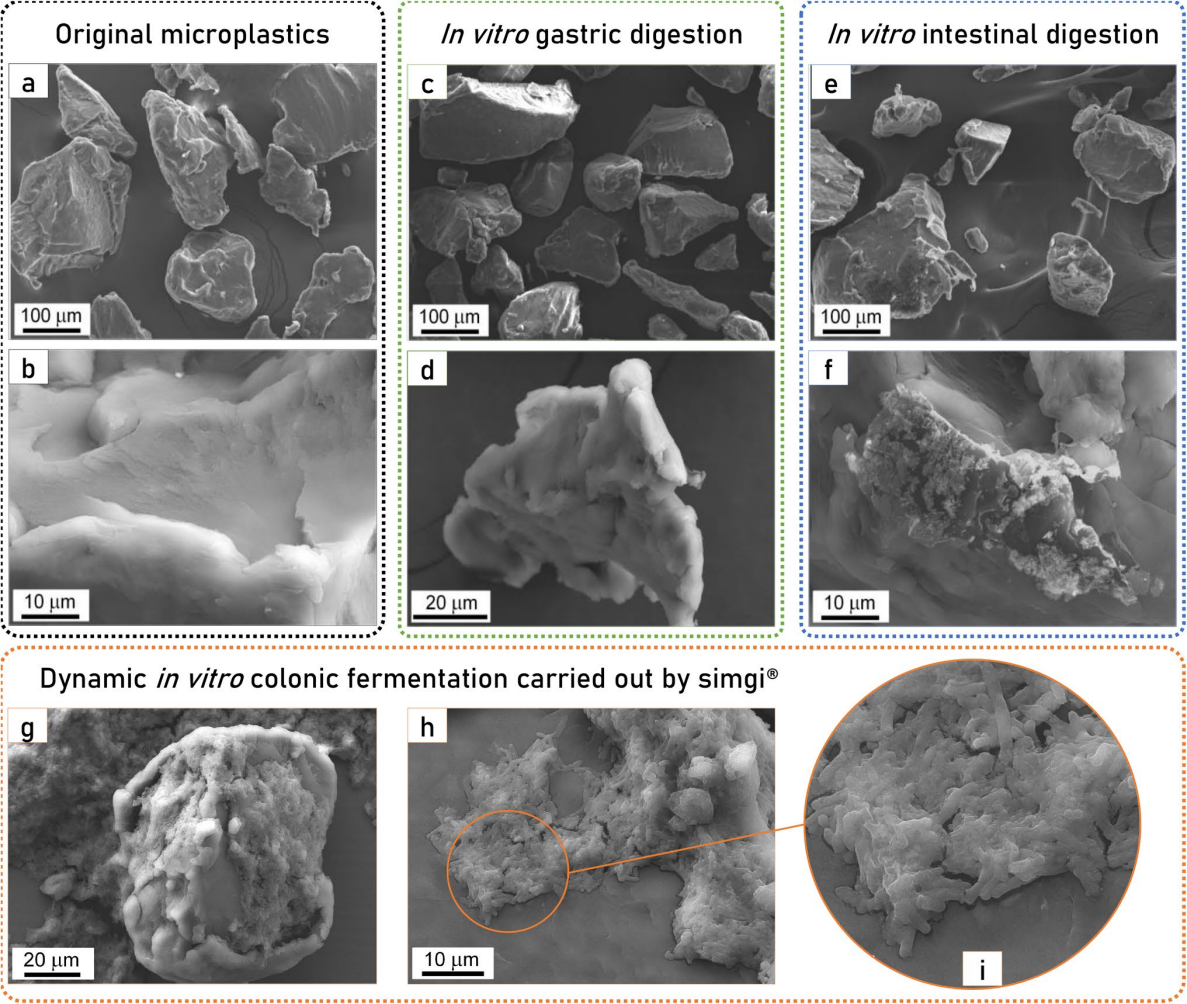
Micrographs by field emission scanning electron microscope (FESEM) of polyethylene terephthalate microplastics: (**a**,**b**) original PET microplastics (MPs), (**c**) and (**d**) PET MPs after in vitro gastric digestion, (**e**,**f**) PET MPs after in vitro gastrointestinal digestion, (**g**–**i**) PET MPs after in vitro gastrointestinal digestion and colonic fermentation.

The original PET MPs average particle size determined by image analysis was 160 µm ± 110 µm and their morphology was irregular. The original PET MPs morphology and surface evidence a surface fracture of brittle material with multiple river marks, which are shown in Fig. 1a. During gastrointestinal digestion and colonic fermentations, the irregular morphology of the MPs is kept, as shown by FESEM micrographs (Fig. 1a–i). After gastric (Fig. 1c,d) and intestinal (Fig. 1e,f) digestions, crystalline and organic matter deposits were observed on particle surfaces, especially after small intestinal digestion (Fig. 1e), but no significant alteration of the polymer surface was observed. However, after dynamic in vitro colonic fermentations, an outstanding change of the PET MPs surfaces is observed (Fig. 1g–i). The higher concentration of organic matter, proceeding from GNM and the colonic microbiota, is deposited on the MPs surface (Fig. 1g,i). Furthermore, Fig. 1i shows in detail microbes on MPs surface.

**Figure 2:**
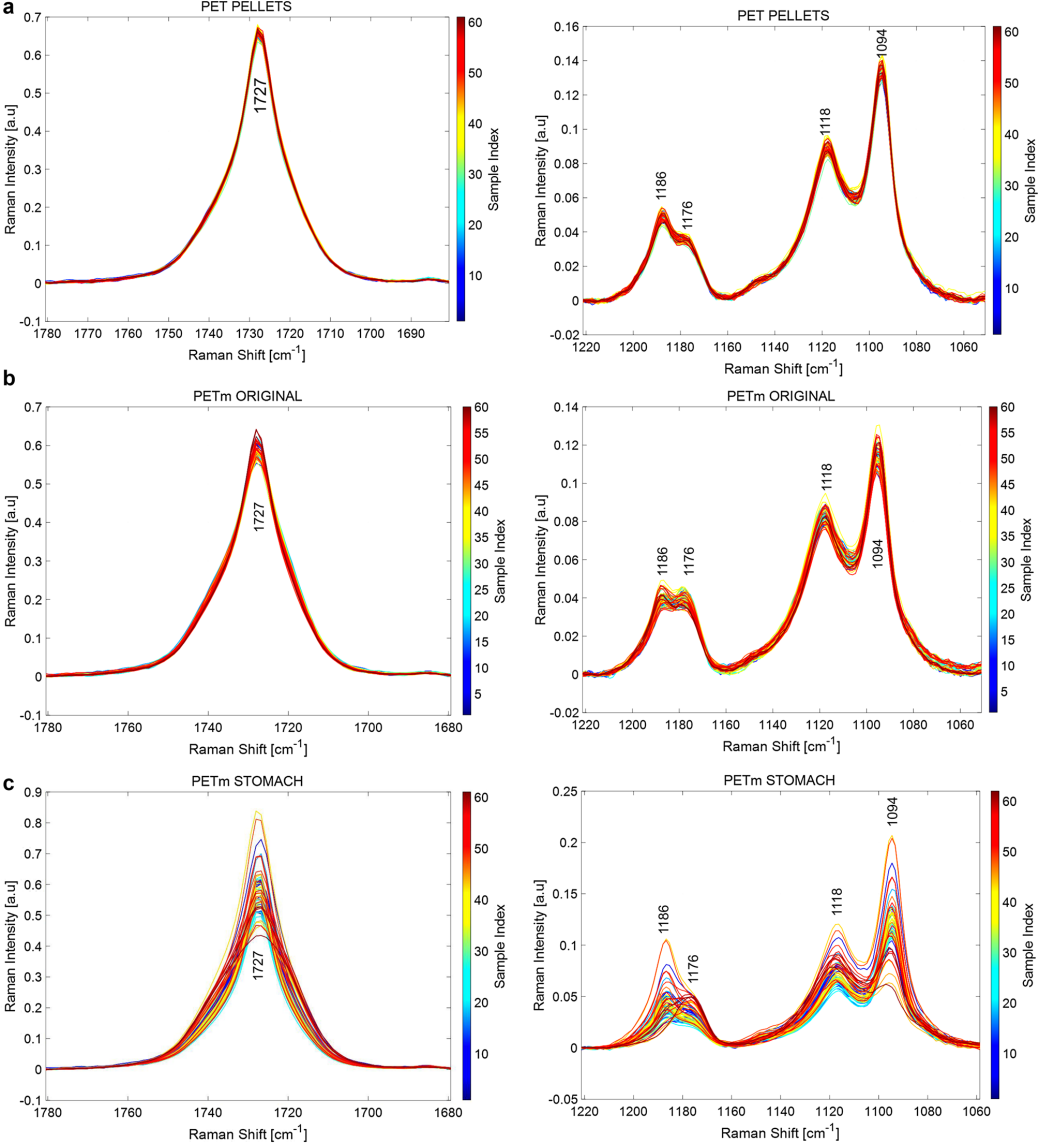

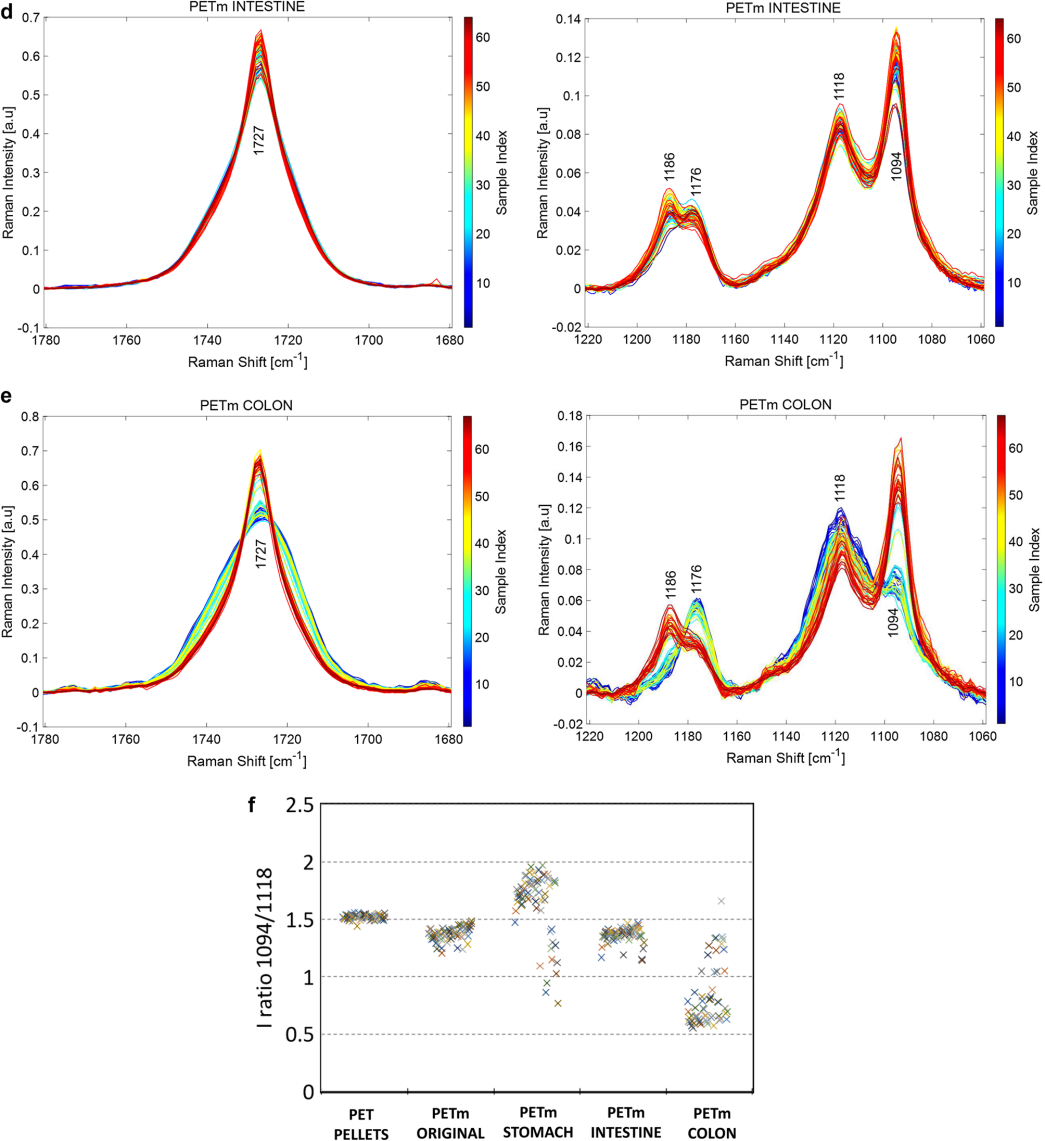
Raman spectra at 60–70 representative points of PET pellets (**a**); original PET microplastics (MPs) (**b**); PET MPs after in vitro gastric digestion (**c**); PET MPs after in vitro gastrointestinal digestion (**d**); and PET MPs after in vitro gastrointestinal digestion and colonic fermentation for volunteer 1 (**e**). (**f**) show the evolution of relative intensity of 1118 modes vs. 1094 cm^−1^ during digestion simulation.

Raman spectra provided further molecular insight on the state of PET MPs. The Raman spectrum of PET presents the characteristic modes of polyethylene terephthalate, in line with known literature^31^. The spectral windows at 1780–1680 and at 1220–1060 cm^−1^ Raman shift shown in Fig. 2 are of particular relevance to assess the crystalline order of PET^32,33^. Figure 2a through 2.e show a progressive amorphization of PET. The Raman mode at 1727 cm^−1^ is associated with carbonyl stretching vibrations and is sensitive to the conformation of the terephthalate groups. In crystalline PET C=O groups are coplanar with an aromatic ring and in trans conformation with each other; these carbonyl groups arrange randomly with respect to the aromatic ring in amorphous PET^34^. A broadening of the band at 1727 cm^−1^ is the consequence of a loss of crystallinity, as illustrated^35^. In addition, the Raman mode at 1186 cm^−1^ associated to the ring ester CCC bending in crystalline PET shifts to 1176 cm^−1^ in amorphous PET^32^. Cryomilling PET pellets to the original PET MPs results in a discrete broadening of 1727 cm^−1^ Raman band of the carbonyl and shift of the 1186 cm^−1^ band of the ester, which are apparent at some spectra of PET MPs. The effect of stomach and intestine digestion is increasingly apparent on the states of PET MPs, where an increasing number of spectra show progressive amorphization. Such a trend is more evident after colonic fermentation, (Fig. 2e). Further to these two Raman modes, the rise in the relative intensity of 1118 vs. 1094 cm^−1^ is also associated with oxidative amorphization of PET^33^. The relative intensity of 1118 modes vs. 1094 cm^−1^ is progressively higher as PET MP evolves along the different digestion stages, as observed in Fig. 2f, which is consistent with the trends uncovered by the 1727 and 1186–1176 cm^−1^ Raman modes. This evolution indicates a trend in the structural degradation of the PET MPs during the gastrointestinal digestion.

### Impact of microplastics intervention on colonic microbiota

**Figure 3:**
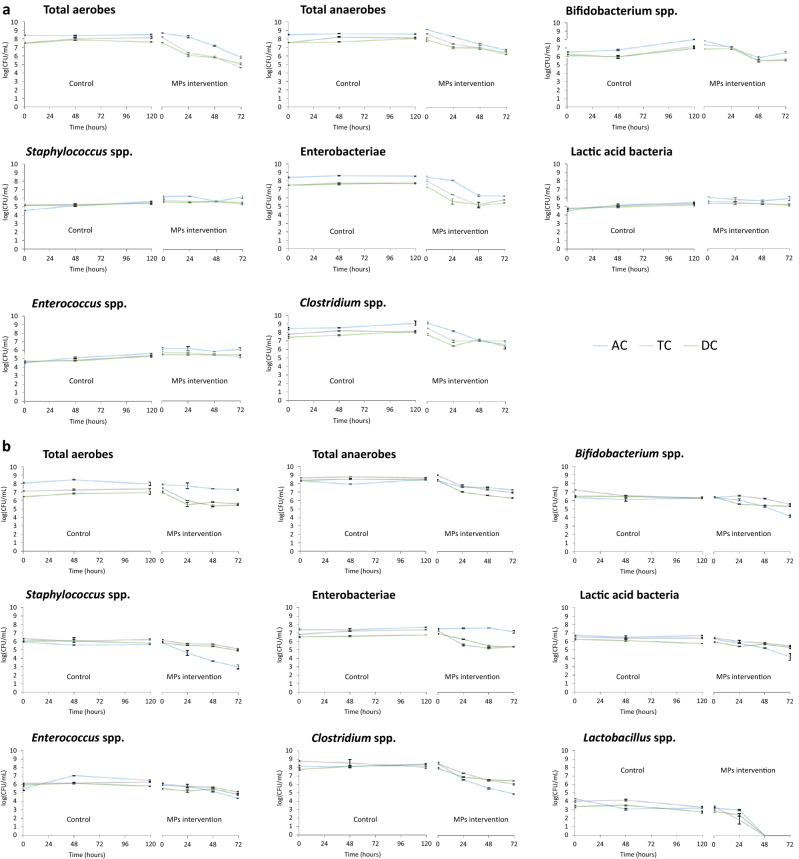
Evolution of the main microbial groups evaluated by plate counting for simgi colonic compartments during control period (0–120 h) and MPs intervention (0–72 h). Figure 3.a show the results for volunteer 1 and Fig. 3.b for volunteer 2. *AC* ascending colon, *TC* transverse colon, *DC* descending colon, *CFU* Colony formatting units.

The effect of PET MPs on the colonic microbiota was evaluated by plate counting as a first approach (Fig. 3). From a microbiological point of view, differences between plate counting values are considered significant for Δ log (CFU/mL) ≥ 1 due to plate counting limitations^22^. Lactic acid bacteria, *Enterococcus* spp. and *Staphylococcus* spp. showed similar plate counting values during 72 h of colonic fermentation for both volunteers, except for *Staphylococcus* spp. values in CA that decreased considerably after MPs exposure. Furthermore, total aerobic and anaerobic bacteria decreased similarly for both volunteers. *Bifidobacterium* spp. and *Clostridium* spp. followed the same decreasing trend, with a significant reduction of viable bacteria in all simgi® compartments. Finally, Enterobacteria group showed a decrease of Δ log (CFU/mL) ≥ 2 in the first 24 h and a slightly lower decrease in the following 24 h in TC and DC compartments for both volunteers, while in AC, the decrease was retarded for volunteer 1 and was not observed for volunteer 2. Other inter-individual differences were observed between the two volunteers, such as the case of *Lactobacillus* spp. not detectable or culturable in volunteer 1, while in volunteer 2 their values decreased after exposure to PMs until 48 h, where they were no longer detectable.

**Figure 4:**
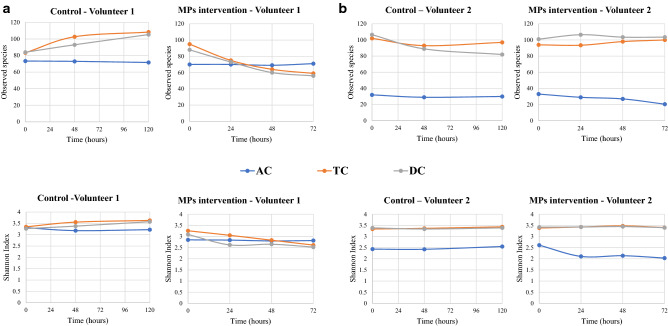
Evolution of the alpha diversity indexes for the different simgi colonic compartments during colonic fermentation. (**a**) show results for volunteer 1 and (**b**) for volunteer 2. *AC* ascending colon, *TC* transverse colon, *DC* descending colon.

Complementarily, 16S rRNA gene sequence analysis and subsequent study of microbial diversity were carried out. Regarding volunteer 1, exposure to PET MPs promoted a drop in the alpha-diversity in terms of Observed species and Shannon index in TC and DC compartments in comparison to control, but not in AC, which values remained stable for the 72 h of colonic fermentation (Fig. 4a).

**Figure 5:**
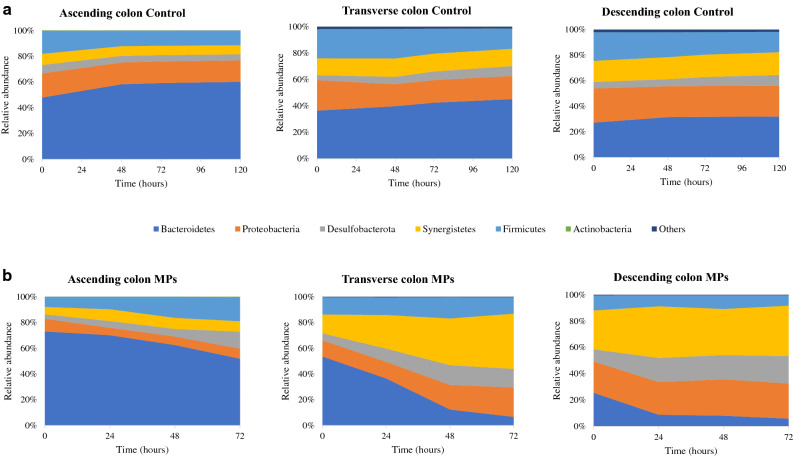
Evolution of the relative abundance at phylum level for the different simgi colonic compartments during colonic fermentation in volunteer 1. (**a**) show results for control period while (**b**) show the effect of MPs intervention. Graphs include the taxa with a relative abundance > 0.5% in at least one of the time points of one simgi colonic compartment.

The phylogenetic analysis revealed differences in the proportions of some taxa after PET MPs intervention in the three colonic simgi® compartments. At phylum level, the differences in the relative abundances were colon compartment-dependent (Fig. 5). In AC compartment, Firmicutes and Desulfobacterota levels increased, while Bacteroidetes proportions decreased. For the TC, Synergistetes, Proteobacteria and Desulfobacterota proportions rose, while the Bacteroidetes relative abundance strongly decreased up to below 10%. In DC, Desulfobacterota members duplicated their proportions in the first 24 h, and Synergistetes levels were also incremented. On the contrary, the relative abundance of Bacteroidetes in DC showed an important decrease of more than 15% in the first 24 h. Then the relative abundance remained stable until 72 h (Fig. 5). The microbiota analysis at genus level corroborated the previously mentioned results, highlighting the decrease in *Bacteroides* and *Parabacteroides* members in all colonic compartments, particularly in TC and DC, where values below 5% were reached (Table 1). Besides, it was also detected a drop in *Alistipes* proportions in all compartments. On the other hand, *Escherichia/Shigella* and *Bilophila* increased their levels in TC and DC, and in the case of *Bilophila* genus, also in AC. The proportions of *Cloacibacillus* also increased in TC and DC, but were maintained in the AC compartment. Finally, *Eisenbergiella*, *Megasphaera* and *Oscillibacter* genus increased their proportions in the ascending colon compartment, supporting the rise detected in the Firmicutes phylum after PET MPs intervention (Fig. 5).

**Table 1:** Relative abundance changes at genus level in each simgi colonic compartment at different times (hours) for volunteer 1. Taxa with a relative abundance > 0.5% are represented.

Genus	Ascending colon				Transverse colon				Descending colon			
	0 h	24 h	48 h	72 h	0 h	24 h	48 h	72 h	0 h	24 h	48 h	72 h
*Alistipes*	2.81%	1.54%	0.46%	0.52%	4.76%	11.39%	4.31%	1.03%	2.47%	0.84%	1.18%	0.88%
*Bacteroides*	57.71%	56.16%	56.17%	46.87%	36.05%	18.36%	5.92%	4.30%	17.87%	7.40%	7.59%	4.99%
*Bilophila*	3.05%	4.74%	5.33%	13.00%	4.85%	9.82%	14.80%	13.89%	8.74%	17.47%	17.62%	20.06%
*Cloacibacillus*	5.66%	9.13%	8.56%	8.03%	14.56%	26.03%	36.22%	42.85%	29.47%	39.20%	34.95%	38.32%
*Coprococcus*	0.29%	0.54%	0.93%	0.64%	0.07%	0.00%	0.00%	0.00%	0.02%	0.00%	0.00%	0.00%
*Desulfovibrio*	0.45%	0.50%	0.56%	0.29%	0.62%	0.51%	0.65%	0.53%	0.49%	0.88%	0.89%	0.77%
*Eisenbergiella*	3.00%	3.09%	5.53%	8.52%	2.19%	5.41%	6.95%	7.20%	1.33%	2.87%	3.61%	2.80%
*Enterobacter*	0.31%	0.30%	0.33%	0.25%	0.44%	0.74%	0.59%	0.35%	0.42%	0.29%	0.13%	0.21%
*Escherichia/Shigella*	7.48%	4.52%	4.95%	5.73%	7.35%	6.13%	10.09%	13.51%	13.33%	15.54%	16.71%	15.56%
*Klebsiella*	1.28%	0.19%	0.13%	0.16%	1.47%	0.13%	0.00%	0.00%	0.71%	0.54%	0.42%	0.42%
*Lachnoclostridium*	0.57%	0.69%	0.49%	0.47%	1.31%	1.10%	1.73%	1.52%	2.20%	2.65%	3.40%	2.82%
*Megasphaera*	1.26%	0.85%	1.75%	4.10%	1.11%	3.55%	4.56%	1.93%	0.72%	0.17%	0.12%	0.04%
*Oxalobacter*	0.00%	0.00%	0.00%	0.00%	0.13%	0.48%	0.96%	0.92%	0.33%	0.51%	0.64%	0.53%
*Parabacteroides*	12.32%	12.05%	5.60%	4.26%	12.00%	4.59%	1.90%	1.22%	4.76%	0.50%	0.26%	0.02%
*Parasutterella*	0.00%	0.00%	0.00%	0.00%	0.66%	1.73%	2.23%	1.98%	0.99%	0.99%	1.03%	0.79%
*Phascolarctobacterium*	1.09%	0.35%	0.25%	0.45%	3.33%	1.45%	0.99%	0.64%	3.60%	1.65%	1.89%	0.64%
*Terrisporobacter*	0.00%	0.00%	0.00%	0.00%	1.03%	0.17%	0.04%	0.04%	0.52%	0.02%	0.02%	0.03%
Others and unassigned	2.72%	5.36%	8.96%	6.68%	8.06%	8.40%	8.06%	8.09%	12.04%	8.50%	9.55%	11.13%

**Figure 6:**
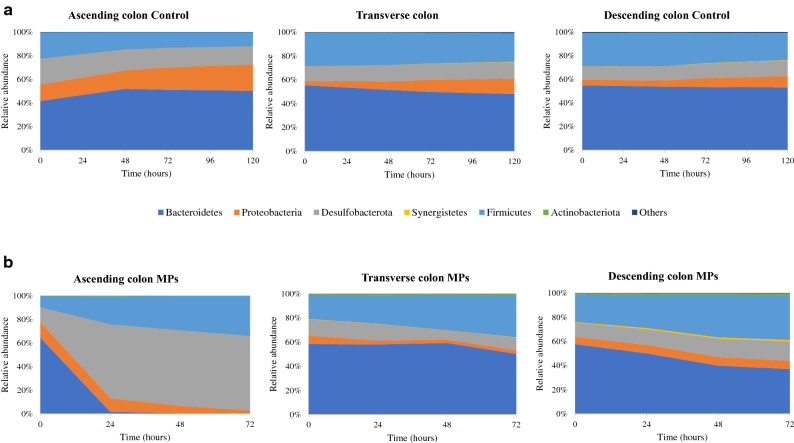
Evolution of the relative abundance at phylum level for the different simgi colonic compartments during colonic fermentation in volunteer 2. (**a**) show results for control period while (**b**) show the effect of MPs intervention. Graphs include the taxa with a relative abundance > 0.5% in at least one of the time points of one simgi colonic compartment.

For volunteer 2, the exposure to PET MPs led to a decrease in the alpha diversity in the AC compartment compared to the control. In contrast, TC and DC compartments seemed to keep their levels stable during the colonic fermentation (Fig. 4b). In this case, the 16S rRNA gene analysis also revealed an increase in AC in Firmicutes and Desulfobacterota levels, while Bacteroidetes proportions decreased (Fig. 6). Besides, an important drop in Proteobacteria levels in AC was detected. For the TC compartment, Bacteroidetes and Proteobacteria relative abundances decreased, whereas Firmicutes members rose. In DC, Firmicutes, Desulfobacterota and Synergistetes increased their proportions during the colonic fermentation, in contrast to Bacteroidetes proportions (Fig. 6). At the genus level, there was a drop in *Bacteroides* in all colonic compartments and a decrease in *Alistipes* in AC and TC (Table 2). *Bilophila* showed a marked increase in AC and DC, while *Lachnoclostridium, Ruminococcus* UCG-002, *Phascolarctobacterium* and *Megasphaera* genus, which increased their proportions in the TC and DC.

**Table 2:** Relative abundance changes at family level in each simgi colonic compartment at different times (hours) for volunteer 2. Taxa with a relative abundance > 0.5% are represented.

Genus	Ascending colon				Transverse colon				Descending colon			
	0 h	24 h	48 h	72 h	0 h	24 h	48 h	72 h	0 h	24 h	48 h	72 h
*Alistipes*	1.03%	0.00%	0.00%	0.00%	0.85%	0.18%	0.23%	0.30%	0.33%	0.59%	1.08%	0.62%
*Anaerotruncus*	0.00%	0.00%	0.00%	0.00%	1.36%	2.07%	1.21%	1.43%	1.84%	0.96%	0.62%	0.33%
*Bacteroides*	63.24%	1.62%	0.56%	0.11%	51.44%	49.42%	52.76%	44.45%	52.12%	39.04%	27.02%	22.18%
*Bilophila*	12.46%	62.64%	64.04%	63.37%	10.11%	10.63%	6.25%	10.59%	9.12%	10.83%	13.96%	15.52%
*Citrobacter*	0.00%	0.00%	0.00%	0.00%	0.84%	0.53%	0.45%	0.61%	1.12%	1.77%	1.83%	1.56%
*Collinsella*	0.00%	0.00%	0.00%	0.00%	0.37%	0.68%	0.31%	0.54%	0.32%	0.24%	0.30%	0.46%
*Coprococcus*	0.00%	0.00%	0.00%	0.00%	0.11%	1.02%	1.12%	1.19%	0.25%	0.48%	0.71%	0.55%
*Desulfovibrio*	0.00%	0.00%	0.00%	0.00%	4.05%	3.56%	1.79%	0.45%	3.41%	3.08%	2.54%	1.73%
*Enterobacter*	0.00%	0.00%	0.00%	0.00%	0.36%	0.37%	0.26%	0.18%	0.56%	0.62%	0.44%	0.52%
*Escherichia/Shigella*	13.41%	11.29%	5.94%	2.46%	3.45%	0.84%	0.73%	0. 84%	1.50%	0.87%	1.10%	1.17%
*Hydrogenoanaerobacterium*	0.00%	0.00%	0.00%	0.00%	0.61%	1.10%	0.75%	0.79%	0.55%	1.12%	1.33%	1.73%
*Intestinimonas*	0.00%	0.00%	0.00%	0.00%	0.57%	0.42%	0.50%	0.69%	0.53%	0.88%	0.83%	0.95%
*Lachnoclostridium*	2.96%	3.23%	4.68%	5.21%	4.77%	5.19%	7.11%	4.36%	4.26%	6.93%	10.43%	10.04%
*Megasphaera*	2.00%	4.34%	3.50%	3.44%	2.69%	3.50%	4.57%	6.99%	4.01%	4.44%	5.08%	6.18%
*Parabacteroides*	0.02%	0.03%	0.00%	0.00%	8.01%	10.25%	8.50%	7.67%	7.37%	13.23%	14.17%	16.45%
*Phascolarctobacterium*	4.31%	15.82%	20.67%	25.03%	6.80%	5.24%	7.72%	9.38%	8.71%	8.41%	10.48%	11.18%
*Ruminococcus* UCG-002	0.00%	0.02%	0.00%	0.00%	0.67%	1.50%	1.82%	3.81%	1.03%	2.49%	3.62%	4.07%
Others	0.58%	1.01%	0.61%	0.38%	2.93%	3.51%	3.91%	5.73%	2.98%	4.02%	4.46%	4.74%

## Discussion

Our study demonstrates that microplastic feeding affects both composition and diversity of colonic microbial communities. To date, the studies on this field have been focused on the effect of MPs on the gut microbial communities of soil animals or mice^14,15,36,37^, and to our knowledge, this is the first report about the modifications and potential effects of microplastics on human colonic microbiota. Moreover, the doses of MPs tested in previous studies with animal models are higher than those detected in edible foods and beverages, but lower than the estimated human daily intake^14,16,36,38–42^. Our approach was to expose human colonic microbiota to a concentration of microplastics closer to reality, so we selected 166 mg/intake of PET MPs, corresponding to the estimated daily intake in humans, according to the study of Senathiraja et al.^3^. We focused our study on the changes during the gastrointestinal digestion and fermentation processes both of PET microparticles’ morphology and structure and of the colonic microbial communities responsible for metabolic bioconversions in the large intestine. For this reason, PET MPs underwent different treatments to simulate the oral, gastric and small intestinal phases of the digestion before getting in contact with previously stabilized human colonic microbiota in the simgi® dynamic simulator, which mimicked the overall impact of digested microplastics on the complex microbial intestinal ecosystem^19,25^.

PET thermoplastic, as well as other polymers materials, show viscoelastic behavior during grinding, which results in a plastic deformation. This inhibited crack initiation, and hence a break-up did not happen. Thus, the PET pellets were grinded in liquid nitrogen, and this allowed cracking them into smaller particles of ca. 60 µm. PET MPs surface evidenced the appearance of brittle fractures with abundant river marks, associated with the compression side during cryogrinding, and few ductile fracture regions. In agreement, the Raman spectra of the MPs suggest a slight loss of crystallinity with respect to the net pellet. Regarding the effect of gastrointestinal digestion and colonic fermentation on PET MPs, FESEM images revealed an evolution of the surface after in vitro intestinal digestion, indicative of a slight interaction with the media, which resulted, according to Raman characterization, with a relative amorphization of the PET structure, but no remarkable morphological changes. PET MPs average size was large enough to avoid disintegration during digestion, allowing us to monitor the MPs at the different intestinal regions. Salt and organic matter deposits were observed on the particle surfaces after gastrointestinal digestion, as previously reported^12^. Crystalline deposits were more abundant, fact that can be related to the increase in salts concentration due to simulated gastric and intestinal fluid addition during gastrointestinal digestion. Besides, some organic deposits, probably forming the so-called (protein-) corona^12^, were also found on PET MPs surfaces at this point, possibly due to the presence of enzymes from the digestion simulation. MPs morphologies show the same appearance after gastrointestinal digestion agreeing with Stock^12^ results, despite particle size differences. It has been suggested that the particles’ size, more than their chemical structure, as well as their possible deformation or degradation, and the protein corona formation during the digestive process are crucial for the bioavailability, and thus for the intestinal uptake rate of the particles and the subsequent toxicological impact and health risks^12^.

After colonic fermentation, PET MPs presented remarkable organic deposits on their surface, which seemed to be colonized by some members of the colonic microbiota, and the surface roughness evolved towards a globular surface. Moreover, the crystalline structure of the PET MPs is affected after the colonic fermentation, which confirms the biotransformation of the polymer by the human digestion process already observed after the gastric step. The FESEM micrographs showed bacteria adhered to the MPs surface and biofilm-like structures were observed in colonic samples, supporting the hypothesis that some members of the colonic microbiota could adhere to MPs surface. In relation to the MPs colonization, different human gut microbial species, such as *Escherichia coli*, *Pseudomonas aeruginosa* and *Staphylococcus epidermidis,* have shown the ability to adhere and even form biofilms on diverse plastic material surfaces such as polyethylene, polypropylene and polystyrene, among others^8,43,44^. Besides, a recent work has shown the adhesion of the gut microbiota of honey bees to the surface of polystyrene MPs^45^. Bacterial communities present on colonic microbiota adhere and colonize the gut mucosa, forming biofilms essential to their cross-feeding relationships, nutrient availability and protection against toxins (i.e. antibiotics), mechanical damage and shear caused by fluid flow^43^. In our study, the possible adhesion of some members of the colonic microbiota to the PET MPs surface could be due to the absence of a mucus layer or intestinal epithelium to adhere to in the simgi®, promoting the formation of biofilms on MPs with the aim to protect themselves and establish their relationships and functions as a community. Another possibility could be the adhesion of some bacterial species able to metabolize and degrade the PET MPs. In this regard, although it has been reported that PET is resistant to biodegradation, various bacterial hydrolases from environmental samples, such as cutinases, lipases, carboxylesterases, and esterases, have been shown to degrade PET to different extents^46^. In general, polymer biodegradation processes include different degrees of material decomposition, which can start with non-enzymatic hydrolysis that promotes fragmentation, followed by assimilation by microorganisms that further involves the enzymatic degradation^47^. In this sense, the *Ideonella sakaiensis* PETase depolymerizes PET, liberating soluble products^48^. Besides, some gut bacterial strains from earthworms have displayed the ability to reduce significantly the particle size of low-density polyethylene^44^. In our work, Raman results showed progressive amorphization of PET MPs during gastrointestinal digestion, possibly related to oxidative amorphization of PET^33^, and more evident after colonic fermentation. These structural changes in the PET MPs particles suggest a potential biodegradation probably driven by colonic microbiota, supporting the existence of an interaction between the colonic microbiota and PET MPs particles. However, although we cannot rule out the presence of PET degrading activities in some specific members of the human colonic microbiota, so far there are no studies or evidence of the existence of human gut bacterial species able to degrade MPs.

Further research is needed to analyze the possible PET MPs colonization by members of human colonic microbiota after ingestion and the mechanisms and functions underlying this adhesion, as well as to explore the possible presence of bacterial activities on human gut microbiome susceptible to biodegrade PET MPs or affect its morphology and structure. Additionally, longer duration experiments should be performed to observe if bacteria from colonic microbiota can degrade plastic over time when faced with a labile C limitation, and test different MPs polymers and sizes, to analyze if the potential degradation could depend on the polymer type and size.

Regarding the effect of the intervention with PET MPs on the colonic microbial communities stabilized in simgi®, plate counts and relative abundances of different bacterial groups revealed changes after 72 h of fermentation. The microbiota underwent a stabilization process prior to the intervention for 14 days followed to a control period of 5 days (120 h), which showed mostly stable microbial populations during this time. So, we assume that without any disturbance, colonic microbiota should maintain their levels and remain stable for the 72 h of PET MPs exposure, so the observed changes are due to the presence of PET MPs.

Bacterial counts showed reductions in total aerobic and anaerobic bacteria, as well as in *Bifidobacterium* spp. and *Clostridium* spp. This suggests that PET MPs and/or their potential resulting constituent monomers exert a negative effect against colonic microbiota, decreasing the levels of total viable bacteria, and to a greater extent of certain microbial groups such as *Bifidobacterium*, *Clostridium* and enterobacteria. Even though the bacterial counts method is widely used in simulators to routinely monitor how the colonic microbiota is evolving during the process, this technique provides information only about the viable and cultivable bacteria, allowing to detect about 20% of the present microbial communities^49^. Hence, it is not the best approximation to deepen into the real microbial changes produced in response to the intervention with PET MPs in simgi®. Accordingly, 16S amplicon sequencing analysis was performed to delve into the microbial communities’ changes, obtaining different trends for the bacterial biodiversity and for different taxonomic levels. The results showed different microbial profiles depending on the volunteer, a fact expected considering gut microbiome interindividual variability (depending on diet, age, lifestyle) and the lack of a *standard* healthy gut microbiome composition^50,51^. Besides, the stabilization process allows the development of the fecal microbiota to colon region-specific microbial communities^25^, but always depending on the initial composition and the relationships established between the microbial communities present. Despite this, and most interesting, exposure to PET MPs promoted similar trends in the two volunteers (depending on the simgi compartment), leading in general to a dysbiotic state, irrespective of the donor, whereas in the control period the proportions and structure of the colonic microbiota remained stable. Regarding biodiversity, PET MPs promoted a decrease in the alpha diversity indices in terms of Observed species and Shannon index. This effect was especially visible for the TC and DC compartments in volunteer 1 and the AC compartment in volunteer 2. The decrease in the alpha biodiversity has been previously reported in animal models after MPs exposure^14,38,39,41,45^, indicating that gut microbiota structure is altered after MPs exposure. Bacteroidetes levels showed an important decrease after PET MPs intervention, a trend observed in the two volunteers and the three colonic compartments of simgi^®^. This result also agrees with those reported in MPs intervention studies with different animal models that detected an important drop in the relative abundance of the members of this phylum^16,38,39,41^. In agreement with other studies^52,53^, our results revealed lower levels of *Bacteroides*, *Parabacteroides* and *Alistipes*, suggesting that MPs could have an important antibacterial effect on these key members of the gut microbiota. *Bacteroides, Parabacteroides* and *Alistipes* species include many important opportunistic pathogens, but, as essential members of a balanced microbiota, they are considered to be health-maintaining. These groups can reinforce the epithelial barrier and ameliorate inflammation by producing anti-inflammatory molecules such as polysaccharide A (PSA), sphingolipids and outer membrane vesicles (OMVs) for the transport of these molecules to the epithelium, and produce antibacterial molecules to prevent the colonization and invasion of exogenous bacteria^54–56^. So, the decrease detected in the proportions of these taxa after PET MPs intervention would mean the decrease, or even loss, of essential groups not only for the maintenance of the correct balance of the gut microbial communities, but also for the intestinal immune homeostasis and barrier function, which imbalance has been associated with systemic and intestinal diseases, such as inflammatory bowel disease (IBD) and irritable bowel syndrome (IBS), among others^54,57^. The increase in the proportions of Firmicutes phyla after MPs intervention observed is a trend previously reported in other animal models such as zebrafish and mice^16,38,39,41^. The changes in the proportions of these two phyla promote an increment in Firmicutes/Bacteroidetes ratio, known to be of significant relevance for the human gut microbiota status and often used as a biomarker in connection with human physiology, being its imbalance related to different metabolic disorders such as obesity and diabetes^58–61^. Moreover, the increase detected in Proteobacteria and Desulfobacterota members (what used to be the class Deltaproteobacteria^62^) also agrees with other studies in different animal models^16,40,42^. In our work this rise was mainly driven by the increase in the proportions of *Escherichia/Shigella* and *Bilophila* genus members in volunteer 1, and also of *Bilophila* in volunteer 2. The increase in *Escherichia/Shigella* proportions was not observed in the volunteer 2 after PET MPs exposure, which could be related to the important relative abundance increment of other old Deltaproteobacteria members, such as *Bilophila* (especially in the AC compartment), and the relative increase of other opportunistic pathogens from Firmicutes phylum, as *Phascolarctobacterium, Lachnoclostridium* and *Megasphaera*, which could exert an inhibitory effect on *Escherichia/Shigella* members growth due to a nutritional advantage or by competitive exclusion. On the other hand, and despite the decrease in bacterial counts of Enterobacteria group, 16S rRNA analysis revealed an increase in the proportions of enterobacteria members, especially in volunteer 1. As it was explained previously, these techniques provide different approaches, not comparable but complementary, being bacterial counts a quantitative approach and the 16S rRNA sequencing a relative abundance result relative to the total number of microorganisms, both cultivable and uncultivable under laboratory conditions. Despite a decrease in the total counts of members of this group is detected, there seems to be an increase in their relative proportion within the microbiota as a whole. A similar effect was also reported by Lu and colleagues, that observed a decrease in α-Proteobacteria levels by qPCR, and an increase in their relative abundance by 16S rRNA sequencing^15^. In a healthy code, Proteobacteria and Desulfobacterota are a microbial signature of inflammation in the gut^63^, and some members belonging to this groups, such as *Escherichia/Shigella* and *Bilophila,,* have been widely associated with a pro-inflammatory effect, as *Bilophila wadsworthia*, that promotes pro-inflammatory TH1 immunity and exacerbates colitis in mice^64^. The rise in the proportions of these pro-inflammatory bacterial groups has also been related to different diseases, such as IBD, colorectal cancer, and coronary artery disease^65–68^. Finally, Synergistetes also revealed an important increase in response to PET MPs in volunteer 1, and less pronounced in volunteer 2, being *Cloacibacillus* genus the main responsible of this effect which has been also® related to pro-inflammatory effects^69–72^.

In summary, our results reveal that the exposure of the human colonic microbiota to PET MPs affected the present microbial communities, as reported for animal models exposed to micro- and nanoplastics^13,37,73^, and even to other nanoparticles^29^. This effect in colonic microbiota might negatively influence the human health. The decrease of microbial groups essential for the correct balance of the gut microbiota and the increase of different pro-inflammatory and disease-related bacterial groups could alter the intestinal homeostasis. This work is the first study that evaluates the potential bidirectional effect of the PET MPs in an in vitro model including gut-microbial fermentation. To date, there are only a few published works focused on this field and none has been carried out in a gastrointestinal simulator, which makes difficult to compare our results. Furthermore, the present work could establish a knowledge basis of the potential PET MPs effects on the bacterial communities present in the human intestine and the possible effect of these microbial populations on PET MPs, which could be useful for future investigations unravelling the effect of MPs on human health.

On the other hand, this study, which considered the impact of PET primary MPs, covered only one of the key possible impact points of MP ingestion on gut microbiota. In the present study a net PET without polymer processing additives was used, however, another critical aspect is the large number of process additives present in polymers, with unsuspected effects on gut microbiota. Moreover, MP ingestion could generate additional risk factors, because MPs coming from the environment could also act as vectors for possible pathogens or contaminants, which could directly or indirectly affect gut microbiota and be related with gut dysbiosis^13,74^*.* Hence, further research is needed to elucidate the effect of MPs intake on the human gut microbiome homeostasis, and thus be able to assess the risk that MPs ingestion through diet has on human health.

## Methods

**Figure 7:**
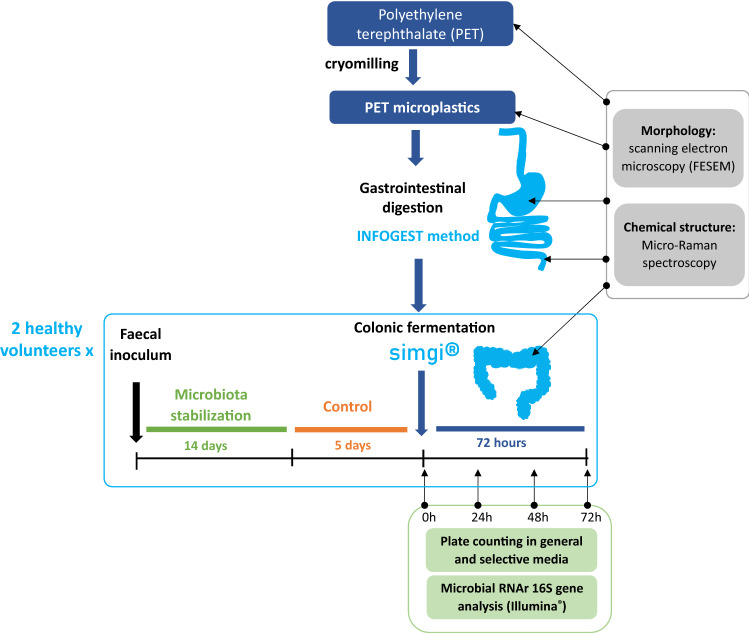
Experimental set-up of the study.

The experimental set-up is showed schematically in Fig. 7.

### Microplastics origin and characterization

Net PET was used without additional processing additives that could distort crystallinity or chemical reactivity. Pure polymer pellets of polyethylene terephthalate (Ramapet N80, Indorama Polymers Europe, Lithuania) were blade milled in liquid nitrogen to obtain PET microparticles. PET MPs and samples from gastrointestinal digestion and colonic fermentations were gold coated (≈200 Å) to prepare samples for size and morphology examination by field emission scanning electron microscopy (FESEM) (Hitachi S-4700, Hitachi, Japan) at 20 keV. Optical analysis was performed in a Multimode Optical Profilometer (Zeta-20, Zeta Instruments, USA). The structural changes of the PET MPs after the gastrointestinal digestion and colonic fermentation were evaluated by micro-Raman in a Renishaw Qontor spectrometer with a 514 nm excitation line laser. The spectral resolution is 1.4 cm^−1^ and every sample was analyzed running a Raman map on at least 60 points to generate representative sampling of the state of PET samples. Each spectrum consisted of 60 accumulations of 1 s with a laser power or 1.5 mW and a 50 × microscope objective. This allows micron-sized space resolution. The total laser power density was low enough to avoid laser damage on the sample during mapping.

### In vitro static gastrointestinal digestions

The selected PET MPs dose for gastrointestinal digestion was 0.166 g/intake, which corresponds to the estimated mean of MPs ingest for humans. Senathiraja et al. assessed that humans may ingest between 0.014 and 0.714 g of MPs daily (0.1 to 5 g per week)^3^. If we consider 4–5 intakes per day, then the intake has a maximum of 0.143–0.179 g.

Gastrointestinal digestions of MPs were performed by INFOGEST method^75^. Briefly, to simulate the oral phase, the MPs were suspended in Milli-Q water and mixed with simulated salivary fluid (SSF)_._ The mixtures were incubated in an orbital shaker for 2 min at 37 °C to simulate oral step, then simulated gastric fluid (SGF) and commercial porcine pepsin were added, providing an enzymatic activity of 2000 U/mL in the final digestion mixture. The pH was adjusted to 3, and the samples were incubated for 2 h at 37 °C. After the gastric stage, simulated intestinal fluid (SIF) was added and the digested samples were set to pH 7. Finally, the intestinal phase was simulated by adding bile salts and pancreatin in order to reach 100 U/mL of trypsin activity and 10 mM of bile salts in the final mixture, which was incubated in the same conditions for 2 more hours. To stop the digestion process, the samples were immediately frozen at − 80 °C (for 24 h) and kept at − 20 °C until posterior use in colonic fermentations.

### In vitro colonic fermentations using simgi®

Colonic fermentations using human colonic microbiota were carried out in simgi® system.

Simgi® is a computer-controlled dynamic simulator of the gastrointestinal tract consisting on five successive reactors able to reproduce gastrointestinal digestion and colonic fermentation^19,25^. In the present study, simgi® system modular design was used to simulate the three regions of the human colon: ascending (AC), transverse (TC) and descending (DC) colon. The reactors were kept at 37 °C and 150 rpm in anaerobic conditions. By automatic addition of 0.5 NaOH and HCl, pH and volumes were maintained at 5.6 and 250 mL for AC, 6.3 and 400 mL for TC, and 6.8 and 300 mL for DC, respectively. Flow rates, compartment volumes, pH, temperature, and transferences were computer-controlled during the experiments.

The system set-up was conducted according to the scheme of Fig. 7. Two independent colonic simulations were carried out, each one with inocula from one healthy volunteer. For each one of the colonic simulations, the process started with the stabilization of the colonic microbiota in the colonic reactors (AC, TC and DC). For this, the three compartments were inoculated with a 20% (w/v) fresh fecal suspension (fecal inoculum) prepared as described in Tamargo et al.^76^, and the system remained in operation for 14 days for the stabilization of the microbiota, as previously described^19^. During the stabilization step, the system was fed every 8 h with 75 mL of Gut Nutrient Medium (GNM) containing arabinogalactan (1 g/L), citrus peel pectin (2 g/L), xylan (1 g/L), potato starch (3 g/L), glucose (0.4 g/L), yeast extract (3 g/L), peptone (1 g/L), mucin (4 g/L), and L-cysteine (0.5 g/L). Once the microbiota was stabilized, a control period of 5 days (120 h) was carried out. Then, each colonic compartment was fed with a single dose of digested PET MPs (0.166 g) and the conditions were kept for 72 h. Samples from all compartments (AC, TC and DC) were specifically collected during the control period (0, 48 and 120 h), before PET MPs feeding (0 h), and after 24, 48 and 72 h PET MPs feeding. Microbial plate count analyses were performed at the time of the sample collection, while other samples were stored at -80 °C until further analyses were carried out. Extra samples from colonic compartments taken to FESEM evaluation were also processed immediately after sample collection. Experimental protocol with the fecal human samples was approved by the Ethics Committee of The Spanish National Research Council (CSIC) being assessed under the internal registration code AGL2015-64,522, and was compliant with the Declaration of Helsinki. Signed informed consent from donors was obtained at the time of enrolment.

### Colonic microbiota analysis

#### Plate counting

Immediately after sampling, ten-fold serial dilutions of AC, TC and DC contents were plated on different types of selective media as described in Tamargo et al.^76^. Briefly: Trypticase Soy Agar (TSA) (Difco™, BD, USA) was used as media for total aerobes plate counting; Wilkins Chalgren agar (Difco™, BD) for total anaerobes; MacConkey agar (Difco™, BD, USA) for *Enterobacteriaceae*; Enterococcus agar (Difco™, BD, USA) for *Enterococcus* spp.; MRS agar (pH = 5.4) (Pronadisa, CONDA, Spain) for lactic acid bacteria; Tryptose Sulfite Cycloserine agar (TSC) (Pronadisa, CONDA, Spain) for *Clostridium* spp.; BBL CHROMAgar Staph aureus (Difco™, BD, USA) for *Staphylococcus* spp.; Bifidobacterium agar modified by Beerens (Difco™, BD, USA) for *Bifidobacterium* spp.; and LAMVAB for specific fecal *Lactobacillus* spp. All plates were incubated at 37 °C for 24 to 72 h in anaerobic conditions (BACTRON Anaerobic Environmental Chamber, SHELLAB, USA), except for BBL CHROMAgar Staph aureus and TSA, which were incubated in aerobic conditions. Plate counting was done by triplicate and data are expressed as log (CFU/mL).

### DNA extractions from colonic samples and Illumina MiSeq sequencing

2 mL samples from AC, TC and DC simgi® compartments were used for DNA extraction using the QIAamp DNA Stool Mini Kit (Qiagen, Hilden, Germany). The V3-V4 region of the 16S ribosomal RNA gene was amplified using forward 5’-CCTACGGGNBGCASCAG-3’ and reverse 5’-GACTACNVGGGTATCTAATCC-3’ primers. The two-step Illumina® PCR protocol was followed to prepare the libraries, and samples were submitted to 2 × 500 bp paired-end sequencing by means of an Illumina® MiSeq instrument (Illumina®, USA). RStudio v.1.3.1093 software (https://www.rstudio.com/) was used to process the files with raw reads from Illumina® instrument. The fastqc files were filtered for reads with low quality and presence of alien DNA using DADA2. DADA2 algorithm was also employed to denoise, join paired-end reads and filter out chimeras in the raw data^77,78^. This algorithm allows the differentiation of even a single nucleotide, leading to the formation of Amplicon Sequence Variants (ASVs). The taxonomic assignment was performed using the naïve Bayesian classifier implemented in DADA2 using Silva v.138 as reference database^79^, with a bootstrap cut-off of 80%. A total of 166 ASVs were found. Biodiversity, expressed in terms of alpha-diversity, was estimated using the ASVs by calculating the Observed, Shannon and Simpson indices through the “Phyloseq” package.

### Colonic microbiota samples treatment prior to FESEM observation

In order to preserve the microbial structure and to better observe the structure of possible biofilms formed on PET surface, the colonic samples were washed twice with PBS 1 × and fixed for 3 h in a solution of 2% glutaraldehyde (Sigma-Aldrich, USA) and 1% paraformaldehyde (Sigma-Aldrich) in PBS 2×. Then samples were in absolute ethanol and conserved at 4 °C until FESEM analysis.
